# There Is No Elevation of Immunoglobulin E Levels in Albanian Patients with Autoimmune Thyroid Diseases

**DOI:** 10.1155/2014/283709

**Published:** 2014-05-14

**Authors:** Hatixhe Latifi-Pupovci, Besa Gacaferri-Lumezi, Violeta Lokaj-Berisha

**Affiliations:** Department of Physiology and Immunology, Faculty of Medicine, University of Prishtina, Deshmoret e Kombit Street, 10000 Prishtina, Kosovo

## Abstract

*Background*. Studies in several ethnic groups reported high incidence of elevated levels of immunoglobulin E (IgE) in patients with autoimmune thyroid diseases (ATD), especially in patients with Graves' disease. *Objective*. To study association between serum levels of IgE and thyroid stimulating hormone receptor antibodies (TRAb) in Albanian patients with ATD. *Material and Methods*. Study was performed in 40 patients with Graves' disease, 15 patients with Hashimoto's thyroiditis, and 14 subjects in the control group. The IgE levels were measured by immunoradiometric assay, whereas the TRAb levels were measured by radioreceptor assay. *Results*. In all groups of subjects the IgE levels were within reference values (<200 kIU/L). Significant difference in mean concentration of IgE was found between two groups of Graves' disease patients, and those with normal and elevated TRAb levels (22.57 versus 45.03, *P* < 0.05). Positive correlation was found between TRAb and IgE only in Graves' disease patients (*r* = 0.43, *P* = 0.006). *Conclusion*. In Albanian patients with ATD there is no elevation of IgE levels. This could be the result of low prevalence of allergic diseases in Albanian population determined by genetic and environmental factors.

## 1. Introduction

The master switch in the regulation of the thyroid gland is the thyroid-stimulating hormone (TSH) receptor (TSHR) [1]. Autoantibodies against thyroid-stimulating hormone receptor (TRAb) are directly involved in the pathogenesis of Graves' disease and autoimmune hypothyroidism [2]. Although the immunoglobulin E (IgE) is the main regulator of allergic reactions, there is evidence which suggests a relationship between autoimmune thyroid diseases (AITD) and allergic diseases. It has been noted that allergic sensitization is more frequent in Graves' disease and allergic seasonality may explain the fluctuation in the onset of Graves' disease [3]; seasonal allergic rhinitis aggravates the clinical course of Graves' disease [4–6]; the serum levels of IgE are significantly elevated in one-third of patients with Graves' disease and lesser reduction in TRAb exists in patients with elevated IgE levels than in patients with normal IgE [7]. Studies in several ethnic groups reported high incidence of elevated levels of IgE in patients with autoimmune thyroid diseases, especially in Graves' diseases [7–13]. Therefore, we conducted ambulatory-based study to investigate whether IgE levels were elevated in Albanian patients with autoimmune thyroid diseases and evaluated potential relationship between IgE and TRAb in patients with Graves' disease and Hashimoto's thyroiditis.

## 2. Material and Methods

### 2.1. Patients

This prospective study included 40 patients with Graves' disease (36 females and 4 males, ages 11–69 yr), 15 patients with Hashimoto's thyroiditis (11 females and 4 males, ages 26–69 yr), and 14 individuals in control group (10 females and 4 males, ages 29–53 yr). Patients included in this study were diagnosed at the Department of Endocrinology, whereas laboratory measurements were done at the Department of Physiology, University Clinical Center, Prishtina, Kosovo. This research was approved by the Faculty of Medicine, Teaching-Science Council, and was conformed to the provisions of the Declaration of Helsinki (paragraphs 11, 13, 15, 16, 20). Informed written consent was obtained from all subjects before inclusion in the study.

The subjects included in this study were untreated patients. Disease diagnosis was based on clinical status, laboratory data—TSH, T3 and T4 levels, and ultrasonographic and histopathologic findings. Basic precondition for inclusion of patients in the study was disease diagnosis based on clinical status and, at least, two of above-mentioned parameters. Patients included in the study were without prescribed therapy for thyroid diseases.

Blood was obtained at their first visit and serum thyroid hormones and TSH were measured at that time. In this study serum concentrations of T3 were measured by total T3 RIA test (reference range: 1.2–2.8 nM) (Immunotech Co., Marseille, France) and T4 was measured by total T4 RIA test (reference range: 60–160 nM) (Immunotech Co., Marseille, France). TSH was measured by total TSH IRMA test (reference range: 60–160 mIU/L) (Immunotech Co., Marseille, France).

### 2.2. Measurement of Autoantibodies and IgE

Sera for measuring autoantibodies and IgE were collected and stored in −20°C until assay. The TRAb concentration was measured by radioreceptor assay (RRA) with DYNOtest TRAK human (B.R.A.H.M.S. Diagnostica, Berlin, Germany). In this study, and as the manufacturer recommends, TRAK human values below 1.0 IU/L were defined as negative and values above 1.5 IU/L as positive. Thyroglobulin autoantibodies (TgAb) concentration was measured by radioimmunoassay (RIA) with DYNOtest anti-Tg_*n*_, (B.R.A.H.M.S. Diagnostica GmbH, Germany). With this method, levels above 60 U/mL were considered positive. Thyroperoxidase antibodies (TPOAb) were determined by radioimmunoassay (RIA) with DYNOtest anti-TPO_*n*_, (B.R.A.H.M.S. Diagnostica GmbH, Germany). Levels of TPOAb above 60 U/mL were considered positive. The IgE concentration was measured by immunoradiometric assay with total IgE IRMA (reference range: 2–200 kIU/L) (Immunotech Co., Marseille, France).

### 2.3. Statistical Analyses

Statistical analyses were performed using two-tailed unpaired Student's *t*-test. *P* < 0.05 was considered statistically significant. Correlation analysis was performed with Pearson's correlation. For statistical analysis the GraphPad Prism 5 software version 5.01 was used.

## 3. Results

### 3.1. Prevalence of Positive TRAb Levels and Mean TRAb Levels in Thyroid Diseases

As stated in materials and methods, sera from 40 patients with Graves' disease, 15 patients with Hashimoto's thyroiditis, and 14 individuals in control group were used for this research. The clinical and laboratory data of patients are shown in Table 1.

In Table 1, data are shown as mean ± SD. In this study, significant difference in prevalence of positive TRAb levels was found between Graves' patients and control group (*P* < 0.001). But prevalence of positive TRAb levels in Hashimoto's thyroiditis did not show significant difference compared to control group (*P* = 0.3). The highest levels of mean TRAb values were found in Graves' patients (mean = 50.32, SD = 30.91) showing significant difference compared to other groups of subjects: Graves' disease versus Hashimoto's thyroiditis and Graves' disease versus control group (*P* < 0.001) (Table 2).

In Table 2, data are shown as mean ± SD. Statistical analyses were performed with Student's *t*-test, *P* < 0.05. Prevalence of positive TRAb levels in Graves' disease is significantly greater than that of control group (*P* < 0.001) but the prevalence of positive TRAb levels is not significant in Hashimoto's thyroiditis compared to control group (*P* = 0.3). The mean TRAb levels are significantly greater in Graves' disease versus Hashimoto's thyroiditis and control group (*P* < 0.001).

### 3.2. Relationship between TRAb Levels and IgE Levels 

In this study, the IgE levels were within reference values in all groups of subjects (Table 2).

Although IgE levels were within reference values, we analyzed if there is any relationship between TRAb levels and IgE levels. First, we grouped all patients in two categories depending on the TRAb levels: patients with normal TRAb levels (negative) and those with elevated TRAb levels (positive). Then we compared mean concentration of IgE among two categories of patients within each group of subjects. Significant difference in mean concentration of IgE was found between two categories of patients with Graves' disease and those with normal and elevated TRAb levels (22.57 versus 45.03, *P* < 0.05). On the other hand, no significant difference in mean concentration of IgE was found among two categories of patients with Hashimoto's thyroiditis and the control group (Table 3).

In Table 3, data are shown as mean ± SD. Data are shown as mean ± SD. Statistical analyses were performed with Student's *t*-test, *P* < 0.05. Significant difference in mean concentration of IgE was found among two categories of patients with Graves' disease and those with normal and elevated TRAb levels (22.57 versus 45.03, *P* < 0.05). No significant difference in mean concentration of IgE was found among two categories of patients with Hashimoto's and the control group.

### 3.3. Correlation between TRAb and IgE

**Figure 1:**
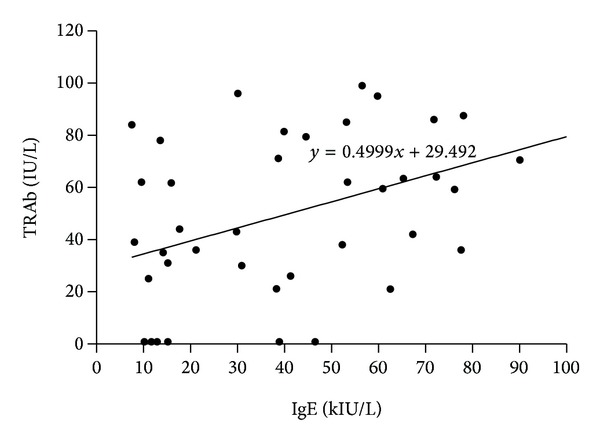
Correlation between TRAb and IgE in Graves' disease patients. Positive correlation between TRAb and IgE in Graves' disease patients and Pearson's correlation coefficient, *r* = 0.43 (*P* = 0.006), with regression line *y* = 0.4999*x* + 29.492.

Among all groups of patients studied, we found a positive correlation between levels of TRAb and IgE only in Graves' disease patients (*r* = 0.43, *P* = 0.006). From the determination coefficient (*r* ^2^ = 0.18) 18% of changes in TRAb level are due to changes in IgE level (Figure 1).

## 4. Discussion

In this study, as expected, the highest prevalence of positive TRAb and the highest mean TRAb levels were found in patients with Graves' disease and our findings support other reports [14–16]. But, in this study, the prevalence of positive TRAb levels was very high in Hashimoto's patients and in the control group. The same implies for TPOAb and TgAb in the control group (Table 2). There are other publications showing positive TgAb and TPOAb in healthy subjects, although being in lower levels [17–19]. Hasse-Lazar et al. found that positive TRAb levels were present in 12.5% of patients with Hashimoto's disease and 4.8% of subjects in control group [15]. According to Trbojevic, the TRAb levels are positive in 50% of patients with Hashimoto's thyroiditis which have TSH levels higher than 5 IU/L [20]. The author Robert Volpe explains this with the fact that the elevated levels of TSH in Hashimotos' thyroiditis could raise the expression of HLA-DR antigens and thyroid antigens which in turn stimulate TRAb elevation [18]. Although the prevalence of TRAb in Hashimoto's patients and healthy subjects was higher than reported by other authors, in this study, mean concentration of these autoantibodies was very low.

In our study, the serum IgE levels in all groups of subjects were within the reference values. Unlike our findings, research carried out on other ethnic groups (Japanese, Chinese, and Korean) showed high prevalence of elevated levels of IgE in two main thyroid autoimmune diseases, particularly Graves' disease [7, 13, 20]. Yamada et al. suggested that an underlying state of autoimmune thyroid diseases may be a permissive factor for IgE elevation [8]. So, given the fact that certain percentage of hyperthyroid Graves' and Hashimoto's thyroiditis patients have allergen sensitization [3] and atopic allergies [8] suggests that allergic states could have an underlying effect which is permissive for thyroid autoimmunity. This could be explained with IL-13 stimulation of B cells to secrete TBII, TSAb, and IgE in Graves' disease patients [13]. Genetic studies in Japanese, Chinese, and Caucasian populations found different data regarding IgE levels in GD [9, 11, 21]. The discrepancy between studies of Caucasian and Japanese populations with regard to IgE synthesis suggests that different genetic factors influence IgE synthesis in different ethnic groups [11].

The absence of elevated level of IgE in Albanian patients with thyroid diseases could be explained with the low prevalence of allergic diseases in Albanian population based on the publications from the International Study on Asthma and Allergy in Childhood (ISAAC) [22–25]. Despite the fact that the above mentioned studies were carried out in Albania, it should be noted that our study was carried out in Kosovo which is inhabited with genetically the same population [26]. Also, environmental factors that influence prevalence of allergic diseases are the same because of geographic proximity and similar social status of ethnic Albanians in Kosovo and Albania.

Although the IgE levels were within reference values in all three groups of subjects, in order to examine if there is any relationship between TRAb and IgE, we grouped patients in two categories: patients with normal TRAb levels and those with positive TRAb. In that case, surprisingly, although being within normal IgE values, we found significant difference in mean concentration of IgE between Graves' disease patients with normal TRAb levels and patients with positive TRAb. Also, there was a positive correlation between TRAb and IgE only in patients with Graves' disease. This suggests that mechanisms for TRAb and IgE synthesis could be linked somewhat in TRAb positive Graves' disease patients. Although there is an association between IgE levels and TRAb, the exact mechanism remains to be determined.

The most striking finding in this study is the absence of elevated IgE levels in Albanian patients with thyroid diseases in Kosovo, which could be a result of genetic and environmental factors associated with allergic diseases, prevalence of which is low in Albanian population. But a small sample may be a limitation for this study. Our data needs to be confirmed with further investigations, such as a study replication on another sample and genetic analyses, to increase confidence in our findings.

## Figures and Tables

**Table 1:** Patients' characteristics and thyroid parameters in various thyroid diseases.

Subject group	Number of patients	Sex (%)		Age (yr)	T3 (nM)	T4 (nM)	TSH (IU/L)
		F	M				
Graves' disease	40	90	10	41 ± 15	4.85 ± 1.81	258.15 ± 108.9	0.49 ± 0.64
Hashimoto's thyroiditis	15	73	27	46 ± 11	0.89 ± 0.34	36.18 ± 22.97	37.15 ± 46.58
Control group	14	71	29	39 ± 6	2.07 ± 0.50	111.82 ± 18.53	1.11 ± 0.64

**Table 2:** Prevalence of positive TRAb levels and elevated IgE levels in various thyroid diseases.

Subject group	TgAb levels (%)	TgAb (U/mL)	TPOAb levels (%)	TPOAb (U/mL)	TRAb levels (%)	TRAb (IU/L)	IgE (kU/L)
Graves' disease	35	135.92 ± 269.4	77.5	1492.97 ± 2413.9	85	50.32 ± 30.9	41.65 ± 26.36
Hashimoto's thyroiditis	93.3	1178.04 ± 1206.5	100	2124.24 ± 1847.6	40	3.63 ± 3.63	33.72 ± 25.79
Control group	7.4	34.35 ± 16.12	21.4	45.92 ± 35.93	21.4	1.15 ± 0.53	37.24 ± 30.63

**Table 3:** Mean serum IgE levels among patients with normal (NEG.) and elevated levels (POZ.) of TRAb.

		TRAb (IU/L)	IgE (kIU/L)
Graves' disease			
TRAb	NEG.	0.9 ± 0.00	22.57 ± 15.86
TRAb	POZ.	59.04 ± 24.64	45.03 ± 26.56
Hashimoto's thyroiditis			
TRAb	NEG.	0.96 ± 0.05	5.4 ± 3.11
TRAb	POZ.	7.63 ± 2.13	38.08 ± 24.92
Control group			
TRAb	NEG.	1.08 ± 0.47	36.38 ± 31.71
