# Corrigendum to “There Is No Elevation of Immunoglobulin E Levels in Albanian Patients with Autoimmune Thyroid Diseases”

**DOI:** 10.1155/2022/9792124

**Published:** 2022-02-21

**Authors:** Hatixhe Latifi-Pupovci, Besa Gacaferri-Lumezi, Violeta Lokaj-Berisha

**Affiliations:** Department of Physiology and Immunology, Faculty of Medicine, University of Prishtina, Deshmoret e Kombit Street, 10000 Prishtina, Kosovo

In the article titled “There Is No Elevation of Immunoglobulin E Levels in Albanian Patients with Autoimmune Thyroid Diseases” [1], citations to 2 related articles were omitted in error [2, 3]. This article [1] is an extended version of the two previously published articles in the print-only journal, *Praxis Medica*, published in Albanian language.

In addition, concerns regarding the statistical analysis were raised by Dr. Driton Vela, University of Prishtina, Kosova. Following a reassessment, it was identified that the Mann-Whitney *U* test should have been used in place of the *t*-test and Spearman's test instead of the Pearson correlation test. The reanalysis yielded some different interim results but does not affect the conclusions of the paper. The corrections are as follows.

In Abstract, the sentence reading “Significant difference in mean concentration of IgE was found between two groups of Graves' disease patients, and those with normal and elevated TRAb levels (22.57 versus 45.03, *P* < 0.05)” should be corrected to “Significant difference in IgE levels was found between two groups of Graves' disease patients with normal and elevated TRAb levels.”

In the Materials and Methods section, the sentence reading “Statistical analyses were performed using two-tailed unpaired Student's *t*-test. *P* < 0.05 was considered statistically significant. Correlation analysis was performed with Pearson's correlation” should be corrected to “Statistical analyses were performed using Mann-Whitney *U* test. *P* < 0.05 was considered statistically significant. Correlation analysis was performed with Spearman's correlation.”

In the Results, the following items should be changed:

1. The text reading “In Table 3, data are shown as mean ± SD. Data are shown as mean ± SD. Statistical analyses were performed with Student's *t*-test, *P* < 0.05. Significant difference in mean concentration of IgE was found among two categories of patients with Graves' disease and those with normal and elevated TRAb levels (22.57 versus 45.03, *P* < 0.05). No significant difference in mean concentration of IgE was found among two categories of patients with Hashimoto's and the control group” should be corrected to “In Table 3, data are shown as Medians (Q1-Q3). Statistical analyses were performed with Mann-Whitney *U* test, *P* < 0.05. Significant difference in IgE levels was found among two categories of patients with Graves' disease with normal and elevated TRAb levels (*U* = 50.500, *P* = 0.049). No significant difference in IgE levels was found among two categories of patients with Hashimoto's and the control group.”
2. The text reading “Then we compared mean concentration of IgE among two categories of patients within each group of subjects. Significant difference in mean concentration of IgE was found between two categories of patients with Graves' disease and those with normal and elevated TRAb levels (22.57 versus 45.03, *P* < 0.05). On the other hand, no significant difference in mean concentration of IgE was found among two categories of patients with Hashimoto's thyroiditis and the control group” should be corrected to “Then we compared IgE levels among two categories of patients within each group of subjects. Significant difference in IgE levels was found between two categories of patients with Graves' disease with normal and elevated TRAb levels (*U* = 50.500, *P* = 0.049). On the other hand, no significant difference in IgE levels was found among two categories of patients with Hashimoto's thyroiditis and the control group.”
3. Table 3 should be replaced with the given table.
4. The text reading “Among all groups of patients studied, we found a positive correlation between levels of TRAb and IgE only in Graves' disease patients (*r* = 0.43, *P* = 0.006). From the determination coefficient (*r*^2^ = 0.18) 18% of changes in TRAb level are due to changes in IgE level (Figure 1)” should be corrected to “Among all groups of patients studied, we found a positive correlation between levels of TRAb and IgE only in Graves' disease patients (*ρ* = 0.392, *P* = 0.012) (Figure 1).”
5. The text within Figure 1 legend reading “Pearson's correlation coefficient, *r* = 0.43 (*P* = 0.006)” should be corrected to “Spearman's correlation coefficient, *ρ* = 0.392 (*P* = 0.012)”.

In the Discussion section, the text reading “In that case, surprisingly, although being within normal IgE values, we found significant difference in mean concentration of IgE between Graves' disease patients with normal TRAb levels and patients with positive TRAb” should be corrected to “In that case, surprisingly, although being within normal IgE values, we found significant difference in IgE levels between Graves' disease patients with normal TRAb levels and patients with positive TRAb.”

## Figures and Tables

**Table 3:** Median serum IgE levels among patients with normal (NEG.) and elevated levels (POZ.) of TRAb.

		IgE (kIU/L)
		Medians (Q1-Q3)
Graves' disease		
TRAb	NEG.	14.05 (11.33–14.80)
TRAb	POZ.	42.95 (17.25–65.80)

Hashimoto's thyroiditis		
TRAb	NEG.	11.20 (5.95–37.95)
TRAb	POZ.	54.35 (34.55–66.03)

Control group		
TRAb	NEG.	28.90 (11.10–50.70)
TRAb	POZ.	48.40 (11.30-)
